# Design and protocol for the Dialysis Optimal Health Program (DOHP) randomised controlled trial

**DOI:** 10.1186/s13063-016-1558-z

**Published:** 2016-09-09

**Authors:** Simon R. Knowles, Chantal F. Ski, Robyn Langham, Emmet O’Flaherty, David R. Thompson, Susan L. Rossell, Gaye Moore, Ya-seng (Arthur) Hsueh, David J. Castle

**Affiliations:** 1 Department of Psychology, Swinburne University, Melbourne, VIC 3122 Australia; 2 Department of Psychiatry, The University of Melbourne, Melbourne, VIC 3010 Australia; 3 Mental Health Service, St. Vincent’s Hospital, Melbourne, VIC 3065 Australia; 4 Centre for the Heart and Mind, Australian Catholic University, Melbourne, VIC 3000 Australia; 5 Department of Neurology, St. Vincent’s Hospital, Melbourne, VIC 3065 Australia; 6 Melbourne School of Population and Global Health, University of Melbourne, Melbourne, VIC 3010 Australia

**Keywords:** Dialysis, Chronic kidney disease, Collaborative therapy, End-stage kidney disease, Psychosocial health, Randomised controlled trial

## Abstract

**Background:**

Chronic kidney disease (CKD) and end-stage kidney disease (ESKD) are serious and growing health problems with enormous impact on psychological and social functioning. Despite high rates of comorbid depression and anxiety in these patient populations, and the adverse impact these have upon treatment adherence, quality of life, social connectedness and healthcare costs there has been little attention focused on the prevention or management of these problems. Thus, our aim was to evaluate the Dialysis Optimal Health Program (DOHP) that adopts a person-centred approach and engages collaborative therapy to educate and support those diagnosed with ESKD who are commencing dialysis.

**Methods:**

The study design is a randomised controlled trial. Ninety-six adult patients initiating haemodialysis or peritoneal dialysis will be randomly allocated to either the intervention (DOHP) or usual care group. Participants receiving the intervention will receive nine (8 + 1 booster session) sequential sessions based on a structured information/workbook, psychosocial and educational supports and skills building. The primary outcome measures are depression and anxiety (assessed by the Hospital Anxiety and Depression Scale; HADS). Secondary outcomes include health-related quality of life (assessed by the Kidney Disease Quality of Life instrument; KDQOL), self-efficacy (assessed by General Self-Efficacy Scale) and clinical indices (e.g. albumin and haemoglobin levels). Cost-effectiveness analysis and process evaluation will also be performed to assess the economic value and efficacy of the DOHP. Primary and secondary measures will be collected at baseline and at 3-, 6-, and 12-month follow-up time points.

**Discussion:**

We believe that this innovative trial will enhance knowledge of interventions aimed at supporting patients in the process of starting dialysis, and will broaden the focus from physical symptoms to include psychosocial factors such as depression, anxiety, self-efficacy, wellbeing and community support. The outcomes associated with this study are significant in terms of enhancing an at-risk population’s psychosocial health and reducing treatment-related costs and associated pressures on the healthcare system.

**Trial registration:**

ANZCTR no. 12615000810516. Registered on 5 August 2015.

**Electronic supplementary material:**

The online version of this article (doi:10.1186/s13063-016-1558-z) contains supplementary material, which is available to authorized users.

## Background

Escalating prevalence and incidence rates of chronic kidney disease (CKD) and end-stage kidney disease (EKSD) are a global challenge [1]. In 2012, around 10 % of Australians (1.7 million) aged 18 and over exhibited measured biomedical signs of CKD; of these, 97 % showed early signs of CKD (stages 1–3) [2]. The overall prevalence of Australians aged 25 and over with ESKD has increased by approximately 20 % from 2005 to 2010 [3]. Dialysis is a challenging experience for most patients, especially in the first year [4–6], with high economic and personal costs to patients and their families, and considerable economic and planning implications for the healthcare system [7]. Commonly reported symptoms of CKD and ESKD include: loss of appetite; insomnia; high blood pressure; and swelling of feet and ankles. Consequently, the disease burden on quality of life is significant [1]. Thus, the patient and family find themselves dealing with multiple disease-associated stressors including balancing the restrictions of this disease within the context of their intimate relationships, families, social networks, treatments, and cultures. Psychological distress, as in other chronic physical diseases, is common in individuals with CKD and ESKD with rates considerably above those in the general population [8–12]. The randomised controlled trial (RCT) described here will adopt a person-centred approach combining collaborative therapy and care coordination to support and improve the psychosocial health and quality of life of those living with CKD and ESKD.

### CKD/ESKD psychosocial interventions

In addition to the increased risk of hospitalisation in ESKD patients with comorbid mental health issues [13], several studies have reported the effect of depression on chronic haemodialysis patient survival to be of similar magnitude to that of medical risk factors [14, 15]. The mechanisms linking depression with survival in this population are unclear but may be related to factors such as treatment adherence, nutritional issues, perceptions of illness, personality, coping styles and increased perception of social support. Adverse biological consequences of the depressed state, including inflammatory, autonomic and neuroendocrine effects also play a role [10]. Psychological distress may also influence a patient’s decision to withdraw from dialysis, accepting palliation, with a recent study finding depression to be significant factor in this decision, particularly where symptoms are apparent in the early stages after commencement of dialysis [14].

A recent review of self-management programs in CKD identified weak evidence that their delivery alone can improve adherence [16]. The authors of that review concluded that life contexts, socioeconomic factors, health literacy and psychological factors, as well as communication with healthcare providers, all contribute to an individual’s adherence to treatment. For example increased psychological distress and poorer communication with healthcare providers was associated with reduced treatment adherence. This review further identified that self-efficacy skill building could potentially improve adherence and therefore be a target for psychosocial interventions [16]. In addition, social processes such as social support can influence psychological changes at an individual level, which may then lead to modified health behaviours. Psychosocial factors are important because they enhance quality of life, in turn slowing the progression of various chronic diseases [17].

The burden and role of depression, anxiety, quality of life and social support in adults with CKD prior to renal replacement therapy was examined in a recent review of the literature [18]. Although the evidence for the impact of psychosocial factors was sparse, the authors did identify that depression and depressive symptoms may independently predict progression to dialysis, hospitalisation and death [18]. However, investigations into the impact of anxiety disorders, social support and quality of life on the clinical course of CKD have received minimal attention. The authors recommended large-scale prospective cohort studies to clarify the burden and prognostic impact of psychosocial factors in this vulnerable population [18].

Although psychosocial interventions are effective in the treatment of anxiety and depression, they have not yet been deployed or evaluated in the ESKD population. To date, there have been no published randomised controlled trials assessing the efficacy of psychosocial interventions for improving depression in ESKD, albeit there is some clinical evidence that these interventions might be effective [19, 20].

### Translating Research, Integrated Public Health Outcomes and Delivery (TRIPOD)

This RCT is part of a larger research program – TRIPOD – which will evaluate our Optimal Health Program (OHP) across three chronic conditions; namely CKD/ESKD, diabetes mellitus and stroke, inclusive of cost-effectiveness analyses. Based on a collaborative therapy framework (CTF; consists of three core components - *education* relating to factors that influence mental health, *coping strategies* that help manage stress and adjustment to illness, and *skills development* to manage stress, illness and long-term optimal health) the OHP was originally developed to support people with mental illness [21]. The initial trial, in an adult mental health service, demonstrated significant improvements in health and social functioning, a reduction in hospital admissions and net cost savings per patient [22]. A key aspect of collaborative therapy is recognising that ‘recovery’ and chronic models of health care are not dichotomous [23]. With the intention of enhancing self-efficacy, self-management, care co-ordination and quality of life, the OHP has been adapted within the broader context of chronic disease. Thus, in the current series of trials our OHP is used to implement this therapeutic framework to enable clinicians and consumers to work systematically towards the achievement of optimal psychosocial health outcomes within mainstream health services. The self-management foundations of the OHP are particularly relevant for adults with ESKD who face the daily challenge of managing various and often simultaneous aspects of their disease such as managing multiple medications, fluid and food intake, ongoing appointments, and monitoring of blood pressure as well as coping with the emotional impact of their care regimen. This protocol describes an RCT to evaluate a dialysis optimal health program (DOHP), a program specifically designed for people living with stage 5 CKD/ESKD.

### DOHP pilot studies

Adaptation of the OHP for people with ESKD was informed by clinical evidence, a review of the associated literature, and pilot data. Two studies, both conducted at St Vincent’s Hospital (Melbourne) were initially conducted that provided (1) information to assist with the development of DOHP and (2) evidence as to the feasibility of DOHP. The first study was a cross-sectional survey of 27 adult patients receiving either haemodialysis or peritoneal dialysis. The aim of this study was to explore the impact of ESKD on individual illness perceptions, coping styles and psychological wellbeing. Key findings indicated that perceptions of illness rather than actual symptoms best accounted for patients’ adaptation to ESKD. Given that the focus of the OHP is to support the patient’s psychological wellbeing, using strategies to improve self-efficacy, empower patients and enhance their self-management skills, these initial findings provided evidence for the suitability of an adaptation of the OHP to those experiencing ESKD and initiating dialysis.

Pilot data was also collated via a RCT (*n* = 12) of the DOHP versus standard care. Patients from a metropolitan and a rural dialysis service were followed over a 12-month period from initiation of dialysis. There were two deaths and one further withdrawal, leaving nine patients who completed the study. Primary outcomes were depression and anxiety. Secondary outcomes included quality of life, treatment adherence, perceived social support, level of function, episodes of psychiatric illness and treatment, medical morbidity and health care utilisation (e.g. outpatient visits; hospitalisations) [10]. An analysis of incidence of depression and anxiety, based on regular face-to-face and phone call assessments using the Mini-International Neuropsychiatric Interview (MINI 6.0) [24], identified incidents of depression and anxiety in the control group only (depression × 3; anxiety × 4). Due to the small size of the trials, conclusions regarding the potential benefit of OHP in relation to the primary and secondary outcomes were not able to be made. However, in relation to the feasibility of the trial, it was clear from participant feedback that the OHP program was associated with high satisfaction, perceived development of skills to effectively manage problems, and would be recommended by others [10].

### Research aims

The aim of this research is to determine whether a dialysis-specific OHP (DOHP) will improve the psychosocial health of dialysis patients, compared to usual care. The primary objective is to identify the impact of DOHP on levels of depression and anxiety in those receiving dialysis. Secondary objectives are to evaluate the impact of DOHP on quality of life, self-efficacy, social and workplace functioning, self-management, and illness perceptions of and coping with ESKD.

A full economic evaluation, namely, cost-effectiveness analysis will be performed to provide further evidence on the efficiency of the DOHP by outweighing health gain and impact on the use of health resources associated with DOHP. The perspectives are from the Australian healthcare system and the patient/family. Quality-adjusted life years (QALYs) will be measured using the Assessment of Quality of Life-6D (AQoL-6D) [25] and European Quality of Life-5 dimensions (EQ-5D-3L) [26]. Process evaluation including focus group interviews will also be conducted with patient and staff participants to assess the effectiveness of the DOHP, implementation, and uptake and service delivery.

## Methods

### General design

This is a prospective randomised controlled trial to evaluate the effectiveness and cost-effectiveness of the OHP specifically adapted for patients receiving dialysis. The DOHP will be delivered as a 9- (8 + 1) week individualised support program using health promotion strategies and will be compared to usual care. Assessments will take place at baseline, 3, 6, and 12 months post-baseline. The study protocol was approved by the St Vincent’s Hospital Human Research Ethics Committee (HREC-A 019/14). An executive steering committee consisting of a nephrologist, a specialist renal nurse, psychologists, psychiatrists, nurses and a health economist oversees project planning, procedures and ongoing data collation.

### Setting

The study will be conducted at the nephrology unit of St Vincent’s Hospital, a large metropolitan teaching hospital in Melbourne, Australia. As of March 2015, the dialysis unit had 290 patients with ESKD receiving dialysis and 190 referred for medical management of CKD with treatment aim of preserving kidney function. The necessary volume of clinical cases and expertise required for this study was established within St Vincent’s nephrology unit in the piloting phase of this research.

### Participants

A minimum of 96 patients initiating dialysis will be recruited into the study. The type of dialysis is haemodialysis, peritoneal dialysis or home dialysis, or collectively known as ‘dialysis’. The following criteria must be met for inclusion in the study: (1) diagnosis of near ESKD confirmed by medical records; (2) expected to commence maintenance dialysis for the first time in the next 3 months or commencement of dialysis in the past 3 months; (3) aged 18 or above; and (4) able to converse in English without an interpreter. Exclusion criteria are: (1) presence of developmental disability or amnestic syndrome impairing their ability to learn from the intervention; (2) participants returning to dialysis following a failed renal transplant; and (3) comorbid serious illness as defined by the treating physician. Individuals who are seeking a mental health professional or taking psychotropic medications will not be excluded from participating.

Power was calculated to detect a medium effect size of *d* = 0.40 using a sample size formula comparing time-averaged differences for continuous outcomes in repeated measurement studies [27]. The power calculations assumed the following: (a) two primary outcome measures (HADS anxiety and depression severity scores); (b) four assessment points (i.e. baseline, 3, 6, and 12 months); (c) a between-repeated measures ρ = 0.70; (d) a study-wide type I error of 0.05 (i.e. a single test α of 0.05 ÷ 5 or 0.01); (f) a type II error rate (β) of 0.20 (power of 0.80); and (g) two-tailed statistical tests. The within-subject correlation (0.70) was based upon a previous OHP trial that demonstrated high correlations over time [22]. The power analysis indicated that 38 participants would be required in each group. Allowing for an attrition rate of 20 %, a total of 96 participants, or 48 in each arm, will be recruited.

### Study procedures

#### Consent

The process of consent will be in accordance with the Declaration of Helsinki. All eligible participants will be fully informed that they are being asked to participate in an RCT. The procedures involved in the study, and the chances of being assigned randomly to one of two groups will be explained verbally and via an information sheet approved by St Vincent’s Hospital Human Research Ethics Committee. A signed consent form will be obtained from each participant. Participants will be made aware of their right to withdraw from the study at any time with no impact on usual clinical care received.

#### Randomisation and blinding

Following the initial screening and gaining of consent, participants will be allocated to either intervention or control group via a computer-generated block randomisation sequence created by an independent person not directly involved in the study. Due to the nature and length of the intervention, it is not possible to blind either patient or investigator to the intervention allocation.

#### Recruitment

**Fig. 1:**
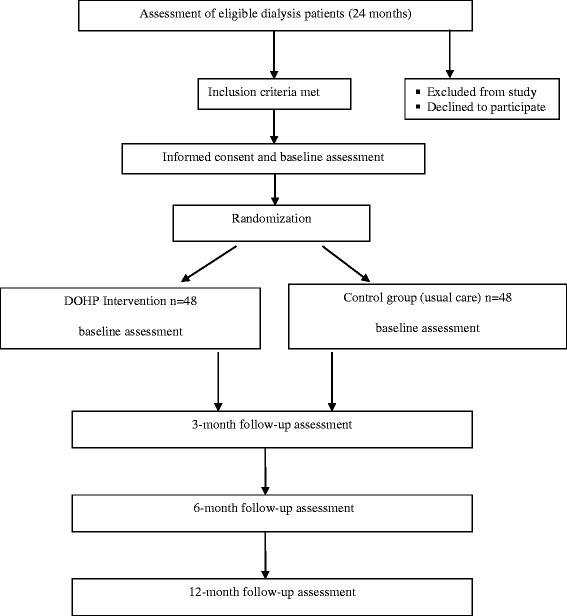
Flowchart of the Dialysis Optimal Health Program (DOHP) randomized controlled trial (RCT)

Potential participants will be identified by dialysis clinical staff based on diagnosis and inclusion criteria and provided with a flyer and verbal explanation. Study fliers, including contact details for the research team, will also be posted online through community organisations. Participants from the community may contact researchers directly to request further information. If agreeable, patients will be informed and formally consented by a research assistant (not associated with the patient’s treatment team) and allocated to either the intervention or control group. CONSORT procedures will be followed throughout the study to ensure the minimum set of recommendations for reporting randomized trials [28]. Participants will be recruited over a 24-month period (see Fig. 1).

### Intervention: DOHP

The DOHP is delivered in nine (8 + 1 booster session) sequential sessions based on a structured workbook. The workbook is central to learning, (i) providing detailed information on the contents of each session and (ii) acting as a health journal where participants can record key dates, appointments, contacts and medication details. Participants are encouraged throughout the program to identify areas of kidney disease or specific concerns upon which they would like to focus. Sessions are approximately 1 hour in duration and held weekly, apart from the ‘booster’ session, which is held 3 months after session 8. Each participant will be allocated to one OHP trained facilitator who will administer the suite of questionnaires, taking approximately 25 minutes to complete, and conduct the intervention. Learning is cumulative with each session designed to build on the previous session including tasks to complete between sessions, such as coping strategies (e.g. breathing exercises).

As the DOHP adopts a holistic collaborative care approach it is not the intention to prevent or treat depression directly, but rather to identify the impact it has on the psychosocial health of patients undergoing dialysis as per the nine sessions as outlined below. In summary, session 1 introduces the DOHP within the six domains of ‘optimal health’; considering the balance of mental, emotional, social, occupational, physical and spiritual needs of a person. This session provides participants with the opportunity to explore and understand their dialysis self-management behaviour from a holistic perspective. Sessions 2 and 3 initiate development of a health plan exploring the implications and potential complications of renal failure and dialysis in terms of strengths and vulnerabilities in session 2, and understanding and monitoring disease impact in session 3 (e.g. the effects of stress on high blood pressure). The focus of session 4 is on metabolic monitoring and medication management (e.g. blood pressure medication and low potassium diet). Session 5 expands the health plan to include key renal failure treatment partnerships and supports in the community and online (e.g. other patients, family, online forums). Session 6 focuses on change enhancement in terms of understanding past events and establishing new proactive avenues for change. The aim of session 7 is goal setting via creative problem solving and planning around the complexities of renal failure and dialysis. Session 8 strategises wellbeing maintenance and sustainability related to the dialysis treatment. The objective of the ‘booster session’ (session 9) is to consolidate progress via reviewing health plans and reflecting on achievements made toward health-related goals. An additional table provides further detail of sessions including behaviour change techniques (see Additional file 1).

A health professional (e.g. nurse, psychologist) trained in the collaborative therapy approach (2-day workshop plus regular supervision and fidelity checks) will facilitate each session. The facilitator will draw on CKD/ESKD-specific information in concordance with individual circumstances. Examples include coping strategies for addressing anxiety related to self-monitoring of blood pressure, availability of community supports, and dealing with the stress of adapting to ESKD and initiating dialysis. Emphasis is placed on the collaboration between facilitator and participant to achieve goals that stem from the participant’s intrinsic concerns and needs. Health promotion is also a major focus, hence the facilitator will encourage participants to identify early warning signs of stress and illness and integrate healthy coping strategies to prevent the build-up of stress. In conjunction with the multidisciplinary team, facilitators will coordinate visits and discuss and arrange referrals to other services depending on participant needs. Further, if at any time during the study a participant identified severe anxiety and/or depression or suicidal ideation they will be contacted by a senior clinician of the research team and where relevant, referred to an appropriate mental health service. Patient participation may be discontinued based upon self-request and/or feedback from the referred treating mental health service. Participants residing in rural and regional areas will have the option of participating in sessions via telephone or Skype (depending on patients’ access to the internet).

### Standard care

Participants randomised into the standard care condition will receive medical care consistent with patients who have ESKD. This will include regular medical appointments with medical staff, diagnostic tests (e.g. blood glucose levels) and access to treatments based upon disease status and recommendations by treating hospital staff.

### Outcome measurements

Primary and secondary outcomes will be assessed at baseline, 3, 6 and 12 months (see Table 1). Primary measures are changes in symptom severity of anxiety and depressive disorders as assessed by the Hospital Anxiety and Depression Scale (HADS) [29]. Secondary measures are: quality of life, as assessed via a disease-specific measure the Kidney Disease Quality of Life (KDQoL) [30] and health-related quality of life as assessed by the Assessment of Quality of Life (AQoL-6D) [25] and European Quality of Life-5 dimensions (EQ-5D-3L) [26] (both scales are highly cited, AQoL with Australian norms allowing comparison); self-efficacy measured by the General Self-Efficacy Scale (GSE) [31], a general sense of perceived self-efficacy in regarding daily hassles as well as adaptation to stressful life events; illness perceptions measured by the Brief Illness Perception Questionnaire (Brief-IPQ) [32], an assessment of cognitive and emotional representations of illness; coping strategies as measured by an abbreviated version of the COPE Inventory [33], the Brief COPE [34]; a 10-item measure of the Big Five personality dimensions [35]; and impact of a person’s mental health difficulties on their ability to function via the Work and Social Adjustment Scale (WSAS) [36]; treatment expectancy and rationale credibility in clinical studies as assessed by the Credibility/Expectancy Questionnaire (CEQ) [37]; perceived acceptability of treatment, assessed using the Treatment Evaluation Inventory-Short Form (TEI-SF) [38]; clinical indices such as albumin and haemoglobin levels and Kt/V (dose of dialysis); and health care utilisation for economic evaluation purposes assessed by the Health Care Utilisation Questionnaire (HCUQ) [39]. Scoring and interpretation of all questionnaires will be undertaken using the recommended published procedures outlined by the relevant questionnaire authors. Adherence to the intervention will be recorded in sessions 2 to 9 by the program facilitators as participants provide feedback on their uptake of the DOHP in the time between each session.

**Table 1:** Primary and secondary outcome assessments and time points for Dialysis Optimal Health Program (DOHP)

Assessment tools	Baseline	3-month	6-month	12-month
Primary outcomes				
HADS (14 items)	X	X	X	X
Secondary outcomes				
AQoL-6D (20 items)	X	X	X	X
Brief COPE (28 items)	X	X	X	X
Brief-IPQ (9 items)	X	X	X	X
CEQ (6 items)		X		
Clinical indices (e.g. Kt/V)	X	X	X	X
EQ-5D-3L (6 items)	X	X	X	X
GSE (10 items)	X	X	X	X
HCUQ (17 items)	X	X	X	X
KDQoL (24 items)	X	X	X	X
TEI-SF (9 Items)				X
TIPI (10 items)	X			
WSAS (5 items)	X	X	X	X

*HADS* Hospital Anxiety and Depression Scale [29], *AQoL-6D* Assessment of Quality of Life-6 Dimensions [25], *Brief COPE* [34] abbreviated version of the COPE Inventory [33], *Brief-IPQ* Brief Illness Perception Questionnaire [32], *CEQ* Credibility/Expectancy Questionnaire [37], *EQ-5D-3 L* European Quality of Life-5 Dimensions-3 Levels [26], *GSE* General Self-Efficacy Scale [31], *HCUQ* Health Care Utilisation Questionnaire [39], *KDQoL* Kidney Disease Quality of Life [30], *TEI-SF* Treatment Evaluation Inventory-Short Form [38], *TIPI* Ten-Item Personality Inventory [35], *WSAS* Work and Social Adjustment Scale [36]

Regarding outcome measurement for the cost-effectiveness analysis the utility measurements of generating quality of life will be assessed using AQoL-6D [25] developed in Australia and the EQ-5D [26]. Regarding cost, health care utilisation of the patient will be collected from the medical records at St Vincent Hospital for inpatient use (patient consent obtained) and by self-administered Health Care Utilisation Questionnaire (HCUQ) [39] for inpatient use other than St Vincent Hospital and all outpatient and community health care use from the patient at baseline and each of the follow-up visits. The sources of the price information are from MBS (Medical Benefit Schedule), PBS (Pharmaceutical Benefit Scheme) and other Australian governmental documents. Both healthcare outcomes and costs will then be compared between participants in intervention and control groups using the incremental cost-utility ratio which indicates the incremental cost per QALY (quality-adjusted life year) of this intervention within the trial period. The estimated long-term (lifetime) impact on cost and effectiveness of the intervention beyond the trial period will be extrapolated using Markov process modelling. The Markov process model will be constructed in reflecting the evolving and progressing of the health state of patients with CKD and ESKD, including the health state of, for example, relapse of depression and anxiety. Appropriate sensitivity analysis for the best and worst scenarios will also be performed based on key variables such as the probabilities of relapse of depression and anxiety to examine the robustness of the cost-effectiveness result.

Due to variability of usual care in the control group, key aspects of standard care will be assessed via responses to the HCUQ [39]. Medical records will also be accessed to confirm diagnostic information, inpatient, outpatient and emergency department visits.

### Program assessment and intervention fidelity

The DOHP facilitators will receive training, a structured manual/protocol and regular supervision (fortnightly with clinical investigators) to discuss any concerns and to ensure standardised delivery of the intervention. Any issues raised by participants can also be discussed at supervision meetings. Sessions will be audio recorded and rated by members of the research team to assure fidelity of the intervention (i.e. does the content of each session accurately reflect the stated content and session plan). Further, an expert of OHP not part of the research team will also randomly sample 10 % of the case notes to ensure sessions conducted comply with OHP session content and procedures. Any variations from the protocol will be fed back to facilitators.

Post-intervention focus groups will be held for clinicians and for participants. The purpose of the focus groups is to gain an in-depth understanding of their experiences of the DOHP, advantages and disadvantages of conducting the DOHP within existing services (for service providers), and suggestions for further components to include or to exclude.

### Statistical analyses

Intention-to-treat analysis will be employed to prevent overestimation of intervention efficacy. Categorical variables will be analysed using chi-squared tests (or Fisher’s exact test for small samples). A mixed-effects model, repeated measures (MMRM) approach will be used to examine the longitudinal profile of the two primary outcome measures at the three time points (3, 6 and 12 months post-baseline). For all MMRM analyses, baseline scores will be used as covariates and the models will include prespecified fixed effects of treatment, clinician and time, in addition to treatment-by-time and treatment-by-clinician interactions. Appropriate adjustments for multiple primary (depression and anxiety) tests will be made, i.e. Bonferroni correction to ensure the risk of type I error is maintained at 5 %. The potential impact of any additional care received by some participants will be assessed in a separate regression analysis using analysis of covariance.

Secondary analyses using analysis of covariance will be conducted to compare change scores during treatment and follow-up points for primary, secondary and process outcomes using the fixed, continuous covariate of baseline score as well as the categorical fixed effects of treatment group, clinician and treatment-by-clinician interactions. Following this, all secondary outcome measures will be assessed, as per above using first a MMRM and then assessing change scores.

Although the attrition rate is not expected to vary by group (intervention vs usual care), we will attempt to identify key predictors of attrition status (i.e. demographic or clinical baseline characteristics) and test for differences between groups. Assuming the data are missing at random, several procedures offer effective approaches that may attenuate attrition. For example, multiple imputation procedures that utilise the expectation-maximization (EM) algorithm with bootstrap estimates of standard errors will be used. A maximum likelihood model with time as a random variable will allow the use of all available data from all assessments, reducing bias and increasing power [40]. Application of these procedures has been shown to provide unbiased estimates, even in the face of substantial missing data [41].

## Discussion

CKD and ESKD are both serious and growing health problems that have enormous impact on social and psychological functioning [4–15]. Despite high rates of comorbid depression and anxiety in these patient populations, as well as disquieting evidence of their potential effects on intervention adherence, quality of life, social connectedness and healthcare costs, there has been little development in the area of prevention or management of these conditions [19, 20]. This trial of an 8 + 1 week psychosocial intervention aimed at improving depression and anxiety with further subsequent psychosocial implications will be the first of its kind undertaken in the CKD transitioning to ESKD with dialysis population.

The DOHP has several strengths, primarily the provision of coordinated care aimed at enhancing the psychosocial health of patients experiencing ESKD. We believe that this innovative trial will contribute to the knowledge of interventions aimed at supporting this patient population and will broaden the focus from symptoms to include psychosocial factors such depression, anxiety, self-efficacy, wellbeing and community supports. In addition, we envisage the quality control component of this trial, via process evaluation, will offer further insight into how the intervention can best be adapted and integrated into the general medical setting.

The outcomes associated with this project are significant in terms of enhancing an at-risk population’s quality of life and psychological wellbeing as well as reducing real treatment-related costs and associated pressures on the renal healthcare system. Furthermore, the proposed RCT will aim to address and reflect the key intent of this major research initiative; a multifactorial, long-term collaborative approach developed via end-user-driven research partnerships that will deliver health benefits to enable Australians with ESKD to age well and productively.

### Trial status

Patient recruitment was ongoing at the time of manuscript submission. Data collection will continue until at least December 2017.

ANZCTR no. 12615000810516.

## Additional file

Additional file 1: This table “DOHP Sessional and Behaviour Change Techniques with Theoretical Framework” provides details of the intervention sessions and related behavioural change techniques. (DOCX 37 kb)

## Abbreviations

AQoL-6D: Assessment of Quality of Life-6 dimensions
Brief-IPQ: Brief Illness Perception Questionnaire
Brief COPE: abbreviated version of the COPE Inventory
CEQ: Credibility/Expectancy Questionnaire
CKD: chronic kidney disease
CTF: collaborative therapy framework
DOHP: Dialysis Optimal Health Program
EM: expectation-maximization
EQ-5D-3L: European Quality of Life-5 dimensions-3 levels
ESKD: end-stage kidney disease
GSE: General Self-efficacy Scale
HADS: Hospital Anxiety and Depression Scale
HCUQ: Health Care Utilisation Questionnaire
KDQoL: Kidney Disease Quality of Life
MINI: Mini-International Neuropsychiatric Interview
MMRM: mixed-effects model, repeated measures
OHP: Optimal Health Program
QALY: quality-adjusted life year
RCT: randomised controlled trial
TEI-SF: Treatment Evaluation Inventory-Short Form
TIPI: Ten-Item Personality Inventory
TRIPOD: Translating Research, Integrated Public Health Outcomes and Delivery
WSAS: Work and Social Adjustment Scale
