# Supplementary material for: Design and protocol for the Dialysis Optimal Health Program (DOHP) randomised controlled trial
Source: Trials. 2016 Sep 9;17(1):447. doi: 10.1186/s13063-016-1558-z (PMC5018180; doi:10.1186/s13063-016-1558-z)
Supplement: Additional file 1: — This table “DOHP Sessional and Behaviour Change Techniques with Theoretical Framework” provides details of the intervention sessions and related behavioural change techniques. (DOCX 37 kb) [file 13063_2016_1558_MOESM1_ESM.docx]

| **Appendix Table 1a**  **OHP Sessional and Behaviour Change Techniques with Theoretical Framework** | | | |
| --- | --- | --- | --- |
| **DOHP Session** | **Behaviour Change Techniques** | **Explanation of BCT** | **Examples of BCT applicable to DOHP** |
| **1. What is Optimal Health?** | **1. Feedback and monitoring**  1.1 Feedback on behaviour  1.2 Self-monitoring of behaviour  **2. Identity**  2.1 Valued self-identity | 1.1 Establish a method for the person to monitor and record their behaviour.  1.2 Establish a method for the person to monitor and record the outcomes of their behaviour.  2.1 Advise the person to write or complete rating scales about a cherished value or personal strength as a means of affirming the person’s identity. | 1.1 Understand the things that you do that have a positive and negative impact on your health.  1.2 Complete the weekly log and sleep log.  2.1 Complete the Optimal Health Wheel rating satisfaction of 6 domains of wellbeing being (1-10) – Domains include: physical, social, emotional, spiritual (values), employment (engagement), intellectual. |
| **2. I Can Do Model (Part 1)** | **3. Self-belief**  3.1 Focus on past success  **4. Shaping knowledge**  4.1 Information about antecedents  4.2 Re-attribution  **5. Goals and Planning**  5.1 Commitment | 3.1 Advice to think about or list previous success in performing the behaviour.  4.1 Provide and discuss information about antecedents social and environmental situations and events, emotions that reliably predict performance of the behaviour.  4.2 Elicit perceived causes of behaviour and suggest alternative explanations.  5.1 Ask the person to affirm or reaffirm statement indication commitment to change of the behaviour. | 3.1 Explore strengths under the following headings: personal qualities, skill and talents, environmental and interests/aspirations.  4.1 and 4.2 Explore vulnerabilities under the following headings: genetic factors, environment, and brain chemistry.  1.9 Develop Health Plan 1. |
| **3. I Can Do Model (Part 2)** | **6. Regulation**  6.1 Reduce negative emotions  **5. Goals and Planning**  5.1 Commitment | 6.1 Advise on ways of reducing negative emotions to facilitate performance of the behaviour –stress management.  5.1 Ask the person to affirm or reaffirm statement indication commitment to change of the behaviour. | 6.1 Explore stressors: positive and negative, stressful situations, early warning signs cumulative stress. Explore strategies and how effective they have been in the past.  5.1 Develop Health Plan 2. |
| **4. Medication and Metabolic Monitoring** | **1. Feedback and Monitoring**  1.3. Biofeedback  **7.Natural consequences**  7.1 Information about health consequences  **6. Regulation**  6.2 Pharmacological support | 1.3 Provide feedback about the body using external monitoring device.  7.1 Provide information about health consequences of performing the behaviour  6.2 Provide/encourage medication information. | 1.3 Encourage completion of metabolic monitoring in collaboration with GP recording test results ongoing.  7.1 Psychiatric Medication Information booklet and specific health information.  6.2 Explore effective use of medication as a strategy to maintain wellbeing. |
| **5. Collaborative Strategies** | **8. Social Support**  8.1 – 8.3 Social support  **9. Antecedents**  9.1-9.4 Restructuring environment  **5. Goals and Planning**  5.1 Commitment | 8.1- 8.3 Provide social support which includes motivational interviewing.  9.1-9.4 Restructuring the environment (physical, social), avoidance and distraction  5.1 Ask the person to affirm or reaffirm statement indication commitment to change of the behaviour. | 8.1- 8.3 and 9.1-9.4 Discuss the collaborative partnerships within the eco map and develop strategies to lessen impact of stress by engaging others for support or to manage difficult relationships or situations.  5.1 Develop Health Plan 3. |
| **6. Change Enhancement** | **5. Goals and Planning**  5.2 Goal setting (behaviour)  5.3 Goal setting (outcome)  5.4 Discrepancy between current behaviour and goal  5.5 Commitment  **1. Feedback and monitoring**  1.4 Self-monitoring of outcome(s) of behaviour  **10. Comparison of outcomes**  10.1 Pros and cons  **11. Covert learning**  11.1 Imaginary reward | 5.2 and 5.3 Set or agree on a goal defined in terms of the behaviour to be achieved  5.4 Draw attention to discrepancies between a person’s current behaviour and previously set outcome goals, action plans.  5.5 Ask the person to affirm or reaffirm statement indication commitment to change of the behaviour  1.4 Establish a method for the person to monitor and record the outcome(s) of their behaviour.  10.1 Advise the person to identify and compare reasons for wanting (pros) and not wanting to (cons) change the behavior.  11.1 Advise to imagine performing the wanted behaviour in a real-life situation followed by imagining a pleasant consequence. | 1.4 Complete a time line activity to explore past strategies and their effectiveness.  5.2, 5.3 and 5.5 Define a change and ask the questions: 1) How important is it for you? and 2) How confident do you feel?  5.4 and 10.1 Complete the decisional balance if the person is ambivalent.  11.1 Anticipate the future and imagine you have achieved your goal. Write down what this might look and feel like. |

| **Session** | **Behaviour Change Techniques** | **Definition** | **Implementation – Session activity within OHP Workbook** |
| --- | --- | --- | --- |
| **7. Visioning and Goal Setting** | **5. Goals and Planning**  5.6 Problem solving  5.7 Action planning  5.8 Review outcome goal(s)  **2. Feedback and monitoring**  2.7 Feedback on outcome(s) of behaviour  **10. Comparison of outcomes**  10.1 Pros and cons  **12. Reward and threat**  12.1 Self-incentive | 5.6 Analyse , or prompt the person to analyse, factors influencing the behaviour and generate or select strategies  5.7 Prompt detailed planning of performance of the behaviour.  5.8 Review outcome goal(s) jointly with the person and consider modifying goal(s) in light of achievement. This may lead to re-setting the same goal, a small change in that goal or setting a new goal instead of, or in addition to the first.  10.1 Advise the person to identify and compare reasons for wanting (pros) and not wanting to (cons) change the behavior.  2.7 Monitor and provide feedback on the outcome of performance of the behaviour.  12.1 Plan to reward self in future if and only if there has been effort and/or progress in performing the behaviour. | 5.6 and 10.1 Brainstorm possible options and ideas. Understand and set a SMARTER goal.  5.7 Step out your goal over the next weeks in your workbook.  5.8, 2.7 and 12.1 Make time to reflect and celebrate the steps taken and achieved. |
| **8. Maintenance and Health Journal** | **5. Goals and Planning**  5.4 Discrepancy between current behaviour and goal  5.5 Commitment  5.8 Review outcome goal(s)  **3. Self-belief**  3.1 Focus on past success | 5.4 Draw attention to discrepancies between a person’s current behaviour and previously set outcome goals, action plans.  5.5 Ask the person to affirm or reaffirm statement  indication commitment to change of the behaviour  5.8 Review outcome goal(s) jointly with the person and consider modifying goal(s) in light of achievement. This may lead to re-setting the same goal, a small change in that goal or setting a new goal instead of, or in addition to the first.  3.1 Advice to think about or list previous success in performing the behaviour. | 5.4, 5.5 and 5.8 Review Health Plans 1, 2 & 3 and Health Journal. Health Plan 1 – Things I need to do every day to maintain my optimal health. Health Plan 2 – Things I need to do when I notice early warning signs and Health Plan 3 – Things I need to do when I experience an episode of illness. Build safety plan.  5.5 and 3.1 Health Journal records contact details, strategies, health plans and also key tools that can be used repeatedly when new challenges occur. |
| **9. Booster** | **5. Goals and Planning**  5.4 Discrepancy between current behaviour and goal  5.5 Commitment  **13. Reward and threat**  13.1 Self-incentive  **3. Self-belief**  3.1 Focus on past success | 5.4 Draw attention to discrepancies between a person’s current behaviour and previously set outcome goals, action plans.  5.5 Ask the person to affirm or reaffirm statement indication commitment to change of the behaviour.  13.1 Plan to reward self in future if and only if there has been effort and/or progress in performing the behaviour.  3.1 Advice to think about or list previous success in performing the behaviour. | 5.4 and 5.5 Review Health Plans 1, 2 & 3.  14.1 Acknowledge achievements  3.1 Celebrate achievements and set future strategies/goals/plans. |

**Adaption from the taxonomy presented by Michie S et al** [Ann Behav Med.](http://www.ncbi.nlm.nih.gov/pubmed/23512568) 2013 Aug;46(1):81-95. doi: 10.1007/s12160-013-9486-6
